# The Role of m5C-Related lncRNAs in Predicting Overall Prognosis and Regulating the Lower Grade Glioma Microenvironment

**DOI:** 10.3389/fonc.2022.814742

**Published:** 2022-03-18

**Authors:** Hongshu Zhou, Ming Meng, Zeyu Wang, Hao Zhang, Liting Yang, Chuntao Li, Liyang Zhang

**Affiliations:** ^1^ Department of Neurosurgery, Xiangya Hospital, Central South University, Changsha, China; ^2^ Hypothalamic Pituitary Research Center, Xiangya Hospital, Central South University, Changsha, China; ^3^ Brain Tumor Center, Xiangya Hospital, Central South University, Changsha, China; ^4^ National Clinical Research Center for Geriatric Disorders, Xiangya Hospital, Central South University, Changsha, China

**Keywords:** lower grade glioma, 5-methylcytosine, lncRNA, tumor microenvironment, prognosis

## Abstract

Glioma is the most lethal primary brain tumor with a poor prognosis and high recurrence rate. Enormous efforts have been made to find therapeutic targets for gliomas. In the current study, we identified m5C-related lncRNAs through Pearson correlation analysis by the criteria |R|>0.5 and p<0.001 in TCGA LGG and CGGA325 datasets. We then established an eight-lncRNA m5C-related prognostic signature (m5C LPS) through lasso cox regression analysis and multivariate analysis. The performance of the signature was confirmed in the CGGA325 dataset and evaluated in differential subgroups divided by relevant clinicopathological characteristics. Patients were then divided into high and low risk groups using risk scores calculated with the signature. Next, we performed GO, KEGG and gene set enrichment analysis (GSEA) and identified the m5C LPS to be related with glioma microenvironment, immune response, EMT, cell cycle, and hypoxia. Correlation of the risk groups with immune cell infiltration, somatic mutation, and CNVs was then explored. Responses to immuno- and chemotherapies in different risk groups were evaluated using submap and pRRophetic R packages respectively. The high-risk group was more sensitive to anti-CTLA4 therapy and to compounds including Temozolomide, Bleomycin, Cisplatin, Cyclopamine, A.443654 (Akt inhibitor), AZD6482 (PI3K inhibitor), GDC0941(PI3K inhibitor), and metformin. We present for the first time a m5C-related lncRNA signature for lower grade glioma patient prognosis and therapy response prediction with validated performance, providing a promising target for future research.

## Introduction

Glioma is the most common type of primary malignant tumor in the central nervous system with a poor prognosis and a high recurrence rate. Lower grade glioma (LGG) refers to a subtype of glioma with WHO grade II or III that presents a less invasive nature and generally better prognosis. Even with surgical resection, chemotherapy and radiotherapy, a large portion of this heterogeneous group of tumors will evolve into high grade glioblastomas. Efforts have been made in developing novel treatment strategies and effective biomarkers for individualized glioma therapy (1). Current biomarkers for disease stratification and individualized treatment include IDH1 mutation, 1p/19q codeletion, MGMT promoter methylation, TP53 and TERT promoter mutation.

RNA modifications regulate multiple cellular processes under biological and pathological conditions. 5-methylcytosine (m5C) is one of the modes of RNA modification mainly accumulating in the vicinity of the 3’UTR, 5’UTR, and near the binding site of Argonaute (2). It confers conserved, tissue-specific and dynamic transcriptional regulation effects including structural stability and metabolism of RNA, tRNA recognition, and stress response (3). m5C modifications are catalyzed by the NSUN family proteins including NSUN1-7 and DNA methyltransferase homologue DNMT2 (4), DNMT1, DNMT3A, and DNMT3B. The m5C RNA methyltransferases display different cellular functions. NSUN1 and NSUN5 modify cytoplasmic ribosomal RNAs; NSUN2, NSUN6 and DNMT2 methylate cytoplasmic transfer RNAs; NSUN3 and NSUN4 install m5C in mitochondrial RNAs (5). Aly/REF export factor ALYREF is reported to be a m5C binding protein facilitating the export of m5C modified mRNAs (6). Depletion of this ‘reader’ protein leads to retention of m5C methylated mRNAs. Another reader of m5C modifications is YBX1 which maintains the stability of target mRNA (7). Aberrant m5C RNA modification is implicated in multiple diseases including cancer (8). Bioinformatics analyses have implicated m5C regulators in prognosis for lung adenocarcinoma (9), hepatocellular carcinoma (10), glioma (7), and others.

Long non-coding RNAs (lncRNAs) are a subgroup of RNAs with over 200 nucleotides that exert non-coding functions. LncRNAs are ideal potential biomarkers for their specificity of expression in different tissues. Furthermore, lncRNAs exert tumorigenic or metastatic effects through different mechanisms including epigenetic modification, post-transcriptional modification, RNA decay or scaffold, and cis-regulation (11). Dysregulation of lncRNAs has been reported to play important roles in glioma genesis. The expression of lncRNAs such as H19, HOXA11-AS, MALAT1, and CRNDE are positively correlated with glioma. MEG3 is highly expressed in normal brain tissue while downregulated in glioma (12). HOXA11-AS is a cell cycle-related lncRNA and a biomarker for glioma prognosis. MALAT1 is reported to be glioma suppressive through attenuating ERK/MAPK-mediated growth and MMP2 mediated invasiveness (13). An NSUN2 methylated lncRNA, NMR, is highly expressed in esophageal squamous cell carcinoma and promotes tumor cell migration and invasion (14). NSUN2 is also reported to target lncRNA H19 and increase its stability through m5C modification. The m5C-modified lncRNA H19 can then be bound to an oncoprotein leading to Myc accumulation, thus exerting oncogenic effects (15). However, there are few studies on the relationship of m5C-related lncRNAs and glioma.

In the current study, we identified eight m5C-related lncRNAs using Pearson correlation analysis and established a m5C related lncRNA prognostic signature (m5C LPS). Performance of the m5C LPS was confirmed in the CGGA325 dataset and evaluated in subgroups divided by several clinicopathological characteristics. Patients were then divided into high and low risk groups using risk scores calculated with the signature. We performed GO, KEGG and gene set enrichment analysis (GSEA) and found that m5C related lncRNAs were related with glioma microenvironment, immune response, EMT, cell cycle, and hypoxia. Correlations of the risk groups with immune cell infiltration, somatic mutation, and copy number variations (CNV) were then explored. Deletions on Chr 9p, 10, 13q and 14q and amplifications on Chr 7, 19, 20 were observed mainly in the high-risk group. CDKN2B, PTEN, EGFR, and IGF2R showed higher CNV rates in the high-risk group. Responses to immunological therapies and chemotherapeutics in different risk groups were evaluated. The high-risk group was found to be more responsive to anti-CTLA4 therapy and to Temozolomide, Bleomycin, Cisplatin, Cyclopamine, A.443654 (Akt inhibitor), AZD6482 (PI3K inhibitor), GDC0941(PI3K inhibitor), and metformin.

## Materials and Methods

### Data Acquisition and Preparation

Transcriptome profiling data of LGG were downloaded from UCSC Xena website (http://xena.ucsc.edu) as a training set. Corresponding clinical data were also retrieved. CGGA325 expression and clinical information data were downloaded from the CGGA website (http://www.cgga.org.cn). Somatic mutation and copy number variations (CNVs) data were obtained from the TCGA website (https://portal.gdc.cancer.gov/). Somatic mutation data was analyzed using the ‘maftools’ R package. Significant copy number variations were detected with GISTIC 2.0 from GenePattern website (https://www.genepattern.org/). Inclusion criteria: TCGA-LGG cases with complete clinical information, corresponding somatic mutation and CNV data were included in the study. CGGA325 data of WHO grade II and III glioma patients with complete clinical information were filtered for use.

### Identification of m5C-Related lncRNAs

LGG transcription data was annotated using Genome Reference Consortium Human Build 38 (GRCh38) to identify lncRNAs and protein coding genes in the TCGA LGG dataset. Regulators of m5C were acquired in previous literature which included NSUN1-7, DNMT1, DNMT2, TRDMT1, DNMT3A, DNMT3B, TET1, TET2, TET3, ALYREF, and YBX1. Next, we performed pearson correlation analysis in the TCGA LGG dataset to identify m5c related lncRNAs by the criteria |R|>0.5 and p<0.001. Univariate cox regression analysis was then performed to acquire m5c-related lncRNAs significantly related to patient prognosis (p<0.05). An identical procedure was performed in LGG samples of CGGA325 dataset and m5C related lncRNAs in the CGGA325 dataset were acquired. Finally prognostic m5C-related lncRNAs were obtained through intersecting results acquired from the TCGA and CGGA datasets.

### Construction of the Prognostic Signature

Lasso regression analysis and multivariate cox regression analysis were utilized to construct a risk score signature in the TCGA dataset following the formula: $Risk\;score=\Sigma_{i=1}^{n}\;({\text{coef}}_{\text{i}}*\text{ex}p_{\text{i}})$ with coef_i_ and exp_i_ representing survival correlation regression coefficient and expression value of each lncRNA, respectively. Risk score was then calculated for each patient in the training set. The median value of risk scores was set as the cutoff to divide the training set into high and low risk groups. Survival analysis was performed and time dependent receiver operating characteristic (ROC) curves were plotted to evaluate the prognostic ability of the signature. Identical analyses were performed in the validation set to verify the signature prognostic value.

Multivariate Cox regression was then performed to establish a nomogram with the m5C-related lncRNAs risk score and clinical features including age, sex, and WHO grade. A calibration plot and concordance index (C-index) were utilized to examine the predictive accuracy of the nomogram using R package ‘rms’. ROC curves and the area under curve (AUC) were also evaluated with R package ‘timeROC’ to review the prognostic ability of the nomogram.

### Functional Analyses

In order to explore differential gene pathways between the high and low risk groups defined by the m5C LPS, Gene Ontology (GO) and Kyoto Encyclopedia of Genes and Genomes (KEGG) pathway enrichment analyses were performed using the ‘clusterProfiler’ R package with BH adjusted p value <0.01. Gene set enrichment analysis was performed with 1000 permutations, p value < 0.05 and false discovery rate < 0.25 using the GSEA 4.1.0 software downloaded from the Broad Institute website (http://software.broadinstitute.org/gsea/).

### Estimation of Immune Infiltration, Somatic Mutation, CNVs and Prediction of Therapy Response

CIBERSORTx was used to estimate the abundance of 22 types of immune cells in the tumor mass (16). CNVs of the high and low risk groups were analyzed with the GISTIC 2.0 module on GenePattern website. The SubMap module on GenePattern website was utilized to evaluate response to immune checkpoint inhibitors of the two risk groups in the TCGA LGG dataset (17, 18) with default parameters.

### Sample Collection

We collected 27 LGG samples from the Department of Neurosurgery, Xiangya Hospital, Central South University. The current study was approved by the Ethics Committee of Xiangya Hospital (No. 201703478). All participants provided informed consent and approval.

### RNA Extraction and rt-qPCR Analysis

Total RNA was extracted using TRIzol Reagent according to manufacturer’s instructions. RevertAid First Strand cDNA Synthesis Kit (ThermoFisher)was used in reverse transcription reaction. ChamQ Universal SYBR qPCR Master Mix (Vazyme, China) and StepOne Real-time PCR systems (Applied Biosystems) were used for rt-qPCR reaction. The experimental condition was set as: 95°C 60s, 95°C 15s, and 60°C 30s for 40 cycles. Reactions were repeated in triplicate for each sample. Expression levels were calculated using 2^-ΔΔCt^ method. Primer sequences are listed in **Supplementary Table S1**.

### Statistical Analysis

Statistical analysis was performed using R software (version 4.0.0). Univariate and multivariate Cox proportional hazards models and lasso regression were used to determine significant prognostic lncRNAs. The Kaplan–Meier curve was used for comparison of overall survival of different subgroups. ROC curve was used to evaluate the predictive efficiency of the m5C-LPS. GSEA 4.1.0 software was used for functional annotation. The rt- qPCR results were analyzed with Student’s t-test. A p < 0.05 was considered statistically significant.

## Results

### Construction of m5c-Related lncRNAs Signature in LGG Patients

**Figure 1:**
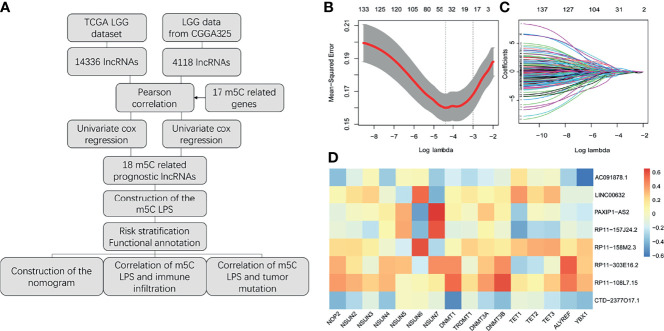
Construction of the m5C related lncRNAs prognostic signature. **(A)** Study flow chart. **(B, C)** Lasso regression analysis for m5C LPS construction. **(D)** Heatmap of the correlations of m5C-related lncRNAs and m5C regulators.

A study flowchart is shown in **Figure 1A**. Gene expression data of 504 LGG samples with complete clinical information was retrieved from TCGA database. We performed Pearson correlation analysis by the criteria |R|>0.5 and p<0.001 acquiring 1121 m5C-related lncRNAs. We then utilized univariate regression analysis (p<0.05) and acquired 607 prognostic m5c-related lncRNAs. Gene expression and clinical data of 172 LGG samples was retrieved from the CGGA325 dataset (**Table 1**). Pearson correlation analysis (|R|>0.5, p<0.001) and univariate regression analysis (p<0.05) yielded 271 prognostic m5C-related lncRNAs. Examining the results from analyses of TCGA and CGGA datasets, 138 common m5C-related prognostic lncRNAs were retrieved. We utilized LASSO Cox regression analysis to filter out prognostic m5C-related lncRNAs yielding 18 lncRNAs (**Figures 1B, C**). We applied multivariate regression analysis to the 18 m5C-related prognostic lncRNAs and constructed a m5C-related LPS consisted of eight lncRNAs (**Table 2**). A heatmap of the correlations between the eight lncRNAs and m5C regulators was plotted (**Figure 1D**).

**Table 1:** Clinical characteristics of cases in TCGA, CGGA and collected glioma samples.

		TCGA LGG (n = 504)		LGG cases in CGGA325 (n = 172)		Clinical samples (n = 27)	
		Cases	Percentage	Cases	Percentage	Cases	Percentage
Age	≥40	267	53.0%	84	48.8%	19	70.4%
	<40	237	47.0%	88	51.2	8	29.6%
Gender	Female	280	55.6%	66	38.4%	12	44.4%
	Male	224	54.4%	106	61.6%	15	55.6%
WHO	II	245	48.6%	98	57.0%	13	48.1%
Grade	III	259	51.4%	74	43.0%	14	51.9%
IDH mutation	Mutant	407	80.8%	127	73.8%	7	25.9%
	Wildtype	97	19.2%	44	25.6%	6	22.2%
	NA			1		14	
1p19q codeletion	Codeletion	163	32.3%	55	32.0%	–	–
	Non-codeletion	341	67.7%	115	66.9%		
	NA			2			
MGMT-nip methylation	Methylated	416	82.5%	85	49.4%	9	33.3%
	Non-methylated	88	17.5%	71	41.3%	4	14.8%
	NA			6		15	

**Table 2:** The eight prognostic m5C-related lncRNAs.

	coef	exp(coef)	se(coef)	z	p
AC091878.1	-1.3674	0.2548	0.3093	-4.421	9.84e-06
LINC00632	0.5022	1.6523	0.2221	2.261	0.02377
PAXIP1-AS2	0.7085	2.0310	0.1832	3.866	0.00011
RP11-157J24.2	0.3221	1.3801	0.1211	2.660	0.00782
RP11-158M2.3	-0.4040	0.6676	0.1013	-3.988	6.67e-05
RP11-303E16.2	0.7230	2.0605	0.1684	4.294	1.76e-05
RP11-108L7.15	-0.8362	0.4334	0.3211	-2.604	0.00922
CTD-2377O17.1	-0.6980	0.4976	0.2523	-2.767	0.00566

### Evaluation and Validation of the m5C-Related lncRNAs Prognostic Signature

**Figure 2:**
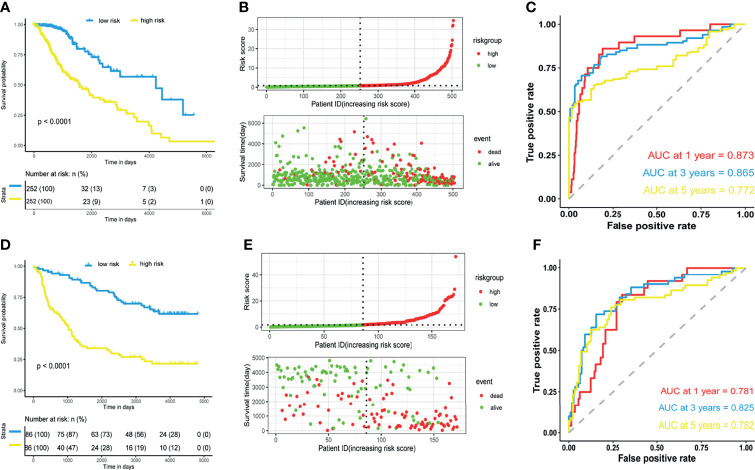
Prognostic performance of the m5C-related lncRNAs signature. **(A, D)** Kaplan-Meier curve showing a better overall survival in low-risk group than the high-risk group in the TCGA and CGGA datasets. **(B, E)** Risk score distribution of patients based on m5C-related lncRNAs signature and survival status in the TCGA and CGGA datasets. **(C, F)** Receiver operating characteristic (ROC) curves showing performance of the signature in predicting 1/3/5-year overall survival in the TCGA and CGGA datasets.

Risk scores of patients in the TCGA dataset were calculated according to the m5C LPS. Patients were divided into high and low risk groups by the median of risk scores. Survival analysis using Kaplan-Meier curves revealed longer overall survival time in the low-risk group (**Figure 2A**). Distribution of risk scores and survival status was plotted in **Figure 2B**, indicating poorer survival in high-risk patients. We then utilized ROC curves to evaluate the prognostic ability of the m5C LPS. One-, three-, and five-year AUC was 0.873, 0.865, and 0.772, respectively (**Figure 2C**), indicating a promising prognostic ability for m5C LPS.

Validation of the m5C LPS was conducted in CGGA325 dataset from which we extracted data of 172 LGG patients. We calculated risk scores with the m5C LPS and divided patients into high and low risk groups in the aforementioned manner. Consistently, survival analysis showed significantly longer OS in the low-risk group than the high-risk group (**Figures 2D, E**). Moreover, one-, three-, and five-year AUC being 0.781, 0.825, 0.782 respectively proved the m5C LPS had a robust prognostic ability in the CGGA dataset (**Figure 2F**). We further validated expressions of the eight lncRNAs in 27 LGG samples collected from the Department of Neurosurgery, Xiangya Hospital of Central South University (**Supplementary Figure 2**). LncRNAs AC091878.1 and RP11-108L7.15 identified as protective factors in the m5C LPS were found to be significantly higher in WHO Grade II tumors while LINC00632 and PAXIP.AS1 presented significantly higher expression in WHO Grade III gliomas.

### Correlation of m5C-Related lncRNAs and Clinicopathological Features

**Figure 3:**
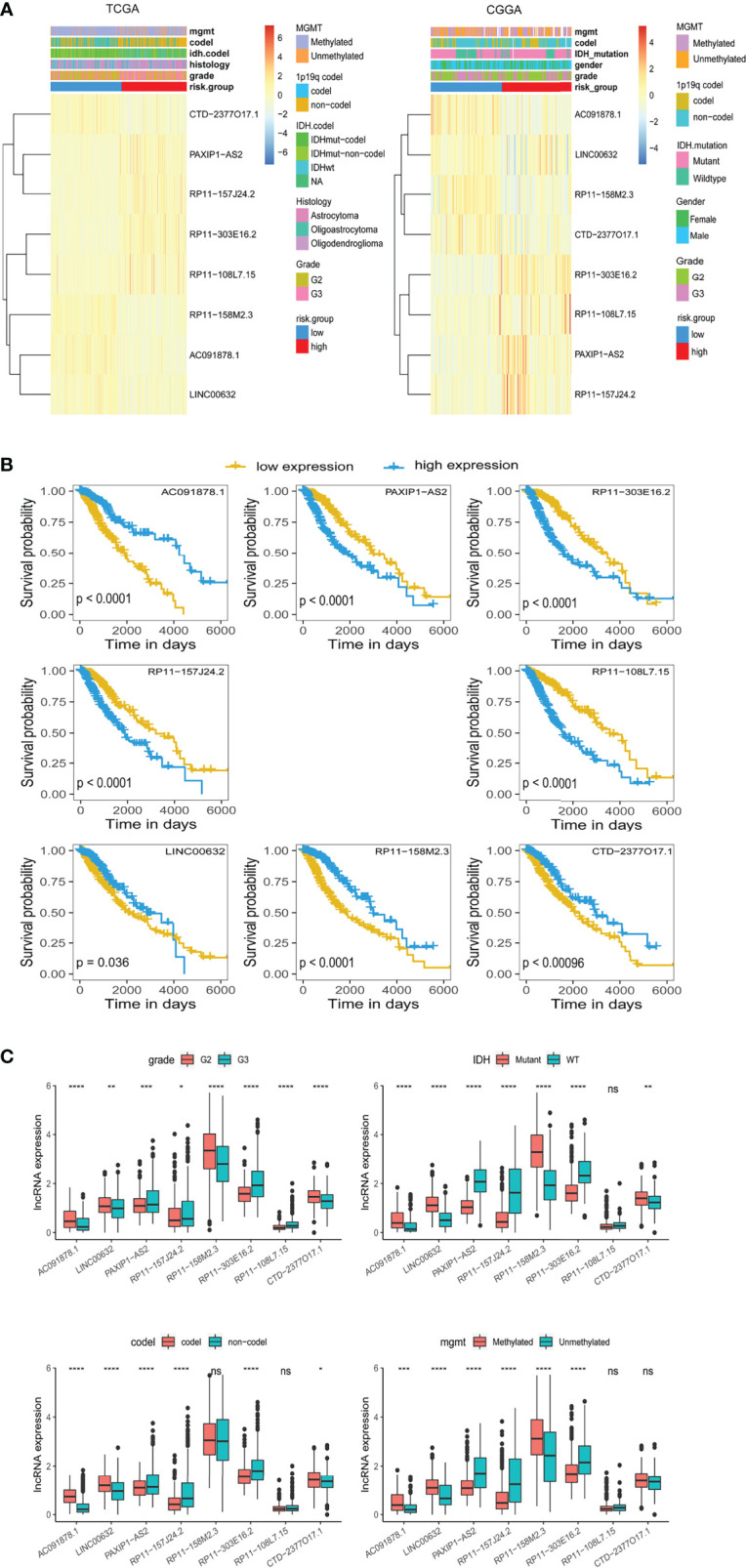
Differential expression of lncRNAs constituting the m5C LPS. **(A)** Heatmaps showing m5C-related lncRNAs expression and clinicopathological features in the TCGA and CGGA datasets. **(B)** Kaplan-Meier curves showing the overall survival of patients grouped by expression of each m5C-related lncRNA in the TCGA dataset. **(C)** Differential expression of the eight m5C-related lncRNAs in WHO grade, IDH, 1p/19q codeletion, and MGMT promoter methylation subgroups. *p<0.5, **p<0.01, ***p<0.001, ****p<0.0001, ns, no significance.

We sought to explore correlation of the m5C LPS with clinicopathological features. MGMT methylation, 1p/19q codeletion, IDH status, and WHO grade were significantly different between the high and low risk groups (**Figure 3A**). Correlation of OS with expression of each lncRNA in the m5C LPS was also investigated through Kaplan-Meier curves (**Figure 3B**). Patients were divided into high and low expression groups according to the expression of each lncRNA. Overall survival was significantly longer in the low expression group for lncRNAs PAXIP1-AS2, RP11-303E16.2, RP11-157J24.2, RP11-108L7.15, while with lncRNAs AC091878.1, LNC00632, RP11-158M2.3, and CTD-2377O17.1 the overall survival was significantly longer in the high expression group, indicating their protective roles in glioma. We then investigated the differential expression of each lncRNA in subgroups divided by WHO grade, IDH mutation, 1p19q codeletion, and MGMT methylation status (**Figure 3C**). Expression of RP11-108L7.15 was only significantly different between WHO grade II and III with no significant difference between IDH, 1p/19q codeletion or MGMT methylation subgroups. Expression of RP11-158M2.3 was significantly different between WHO grade, IDH and MGMT methylation subgroups. Significant differences were also observed in the expression of CTD-2377O17.1 between WHO grade, IDH and 1p19q codeletion subgroups. The other lncRNAs from the m5C LPS all showed significant differences in subgroups of WHO grade, IDH, 1p19q codeletion and MGMT methylation, indicating their differential roles in gliomas.

**Figure 4:**
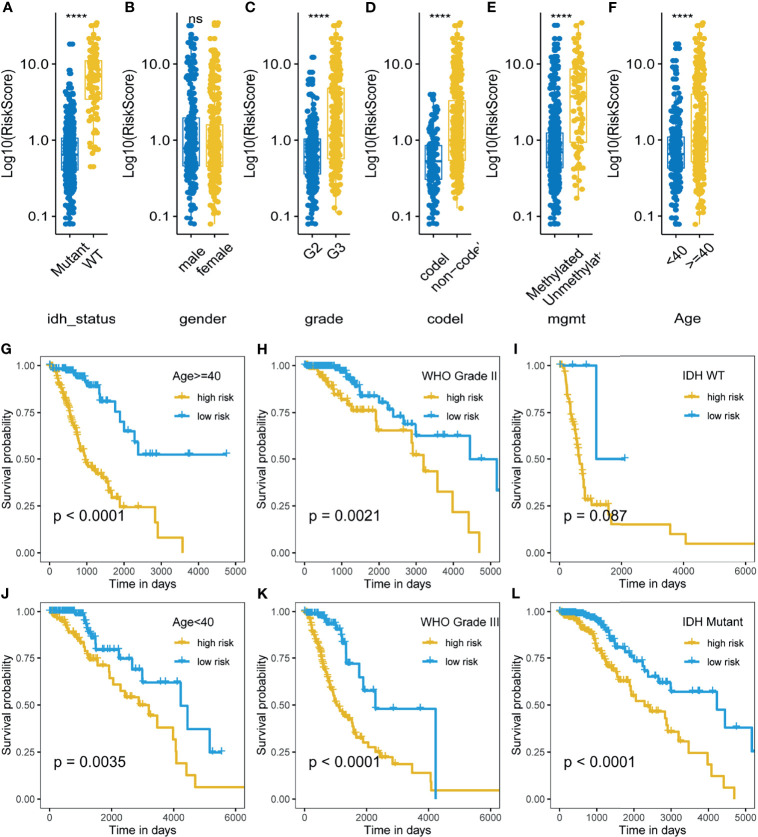
Correlation and prognostic performance of the m5C LPS. **(A–F)** Different risk scores between patients grouped by clinicopathological features including IDH mutation status, 1p/19q codeletion status, MGMT methylation status, WHO grade, age and gender. **(G–L)** Kaplan-Meier curves showing stable performance of the signature in differential subgroups of LGG patients including age, WHO grade, and IDH status. ****p<0.0001, ns, no significance.

We also investigated the distribution of m5C LPS risk scores between different clinicopathological subgroups (**Figures 4A**–**F**). Risk score was significantly higher in the following subgroups namely, IDH wild type, WHO grade III, 1p19q non-codeletion, MGMT promoter unmethylated, and age > 40. Survival analysis was also performed in each subgroup *via* KM curve and proved the m5C LPS had robust prognostic ability in each subgroup of different clinicopathological characteristics (**Figures 4G–L**).

### Construction of the Nomogram

**Figure 5:**
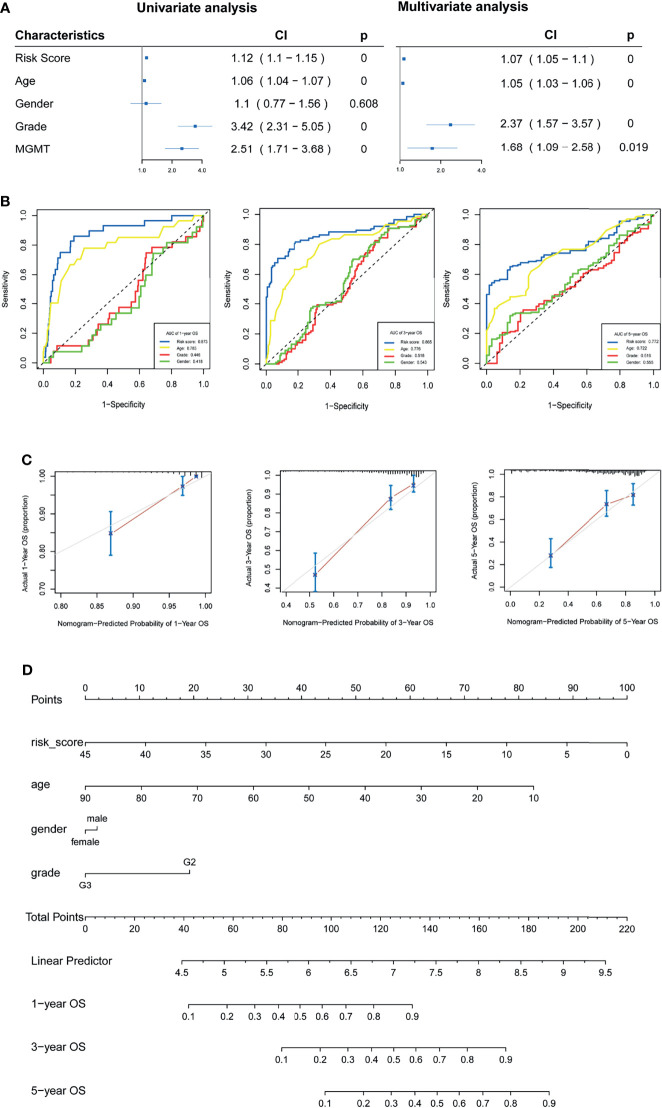
Verification of the m5C LPS and construction of the nomogram. **(A)** Forest plots showing the results of univariate and multivariate Cox regression analyses on the prognostic performance of the risk score and clinicopathological features. **(B)** ROC curves showing prediction performance of the risk score and clinicopathological features. **(C)** Calibration curve of the nomogram for 1,3,5-year OS. **(D)** Construction of the nomogram using risk score and clinicopathological features.

Univariate and multivariate analyses were used to investigate the prognostic value of the m5C LPS (**Figure 5A**). In univariate analysis, a hazard ratio (HR) of 1.12 (CI: 1.1-1.15) with p value <0.01 indicated that the m5C LPS risk score is a prognostic indicator. In multivariate analysis, the risk score presented an HR of 1.07 (CI: 1.05-1.1) with a p value <0.01 indicating the risk score to be a prognostic indicator independent of age, grade and MGMT status. An ROC curve was implemented to evaluate the specificity and sensitivity of prognostic indicators and manifested a higher AUC for the risk score than other clinicopathological features (**Figure 5B**). We then constructed a nomogram with the m5C LPS risk score and other clinicopathological features (**Figure 5D**). A C-index of 0.823 and conformity in nomogram-predicted and actual 1, 3, 5-year OS of patients (**Figure 5C**) indicated a promising prognostic ability.

### Functional Annotation of Low and High-Risk Groups

**Figure 6:**
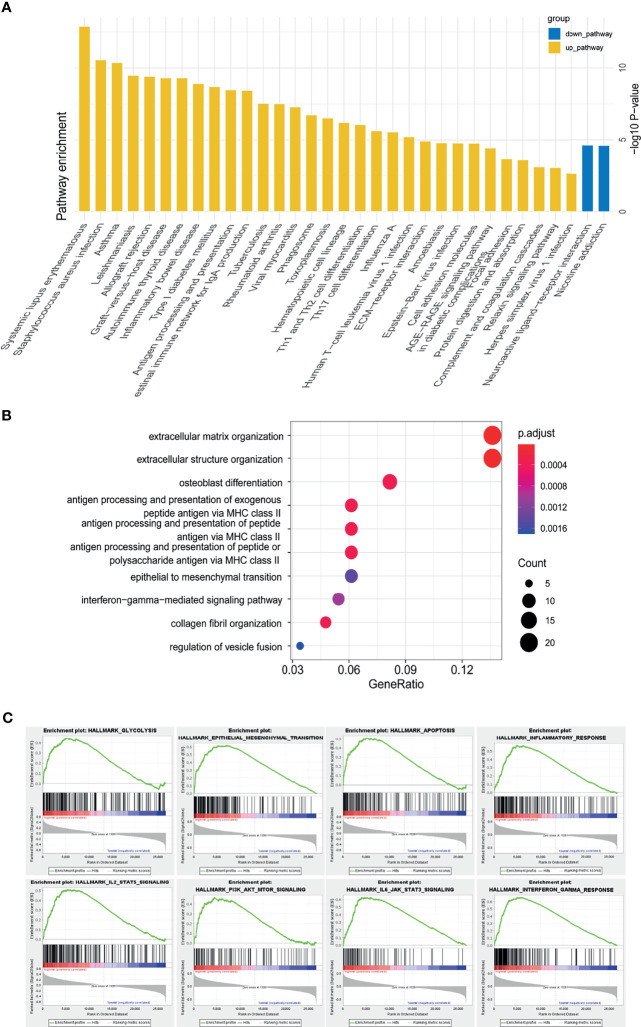
Functional annotation of risk groups identified through the m5C LPS. **(A)** KEGG pathway enrichment analysis between high and low risk groups. **(B)** Dotplot of GO biological processes. **(C)** GSEA analysis showing pathways enriched in the high-risk group.

We performed GO, KEGG and GSEA analyses to identify pathways activated in the high-risk group. Th1, Th2 and Th17 cell differentiation, ECM-receptor interaction pathways were enriched in the high-risk group. GO showed extracellular matrix organization, epithelial to mesenchymal transition (EMT), and interferon gamma mediated signaling pathway were enriched in the high-risk group. GSEA analysis identified glycolysis, epithelial to mesenchymal transition (EMT), inflammatory response, interferon gamma response, PI3K-AKT-mTOR signaling, IL6-JAK-STAT3 signaling, IL2-STAT3 signaling, E2F targets, interferon alpha response, G2M checkpoint, and hypoxia were positively correlated with high-risk group (**Figures 6A–C**). The analyses indicated m5C related lncRNAs were possibly related with the glioma tumor microenvironment, immune response, EMT, cell cycle, and hypoxia.

### Correlation of m5C-Related lncRNAs With the Glioma Tumor Microenvironment and Response to Immunotherapies

**Figure 7:**
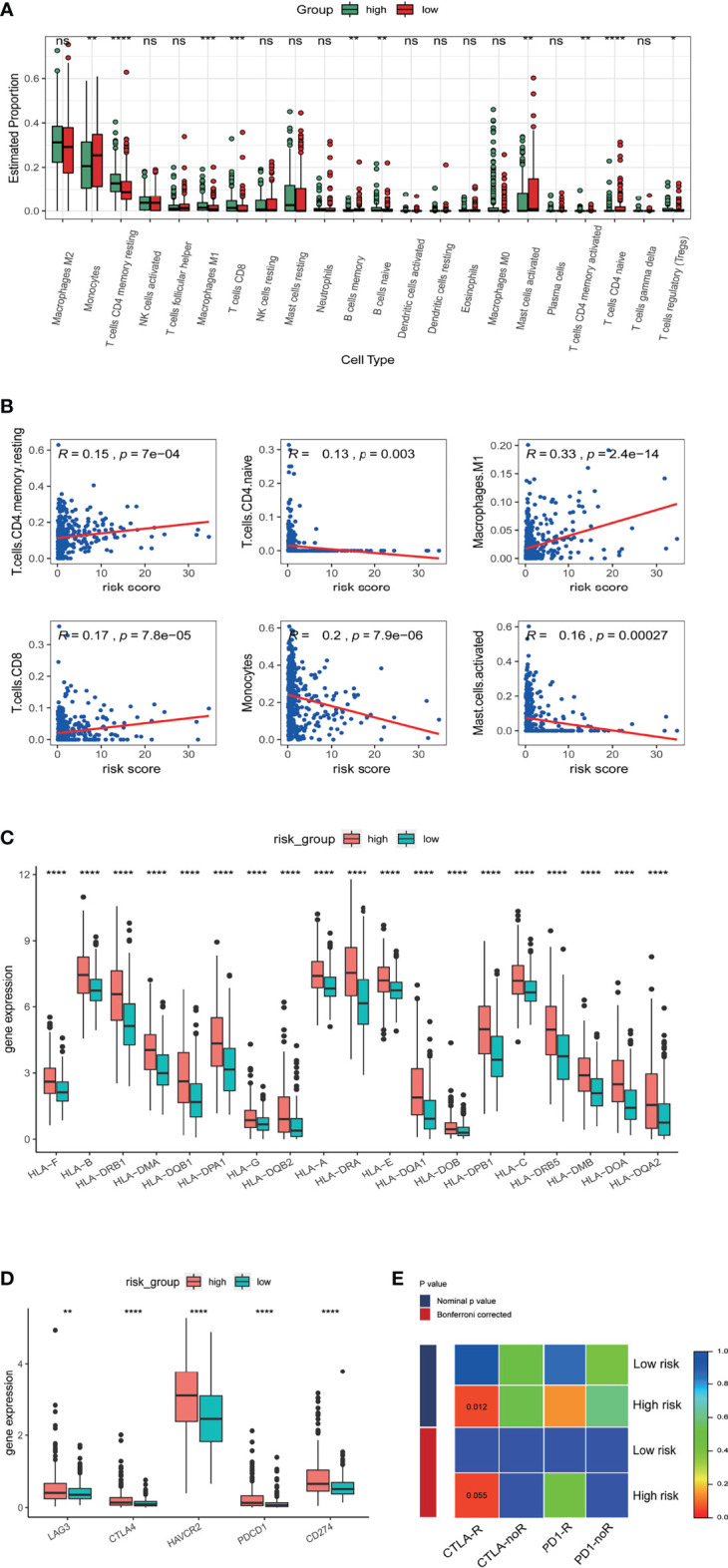
Correlation of the risk score with immune cell infiltration and prediction of responses to immune checkpoint inhibitors. **(A)** Estimated proportion of 22 tumor infiltrating immune cells in high and low risk groups. **(B)** Correlation of risk score with proportion of six tumor-infiltrating immune cell types. **(C)** The expression levels of HLA family genes in low- and high-risk groups in TCGA database. **(D)** The expression levels of CD274 (PD-L1), CTLA4, HAVCR2, LAG3, and PDCD1 (PD1) in low- and high-risk groups in TCGA database. **(E)** Sensibility of patient responses to PD1 and CTLA4 inhibitors in different risk groups. *p<0.5, **p<0.01, ***p<0.001, ****p<0.0001, ns, no significance.

We next investigated the correlation of m5C related lncRNAs with glioma immune infiltration using CIBERSORTx. An overall high proportion of M2 macrophages, monocytes, CD4 memory T cells, activated and resting mast cells in LGG is shown in **Figure 7A**. Monocytes, memory B cells, activated mast cells and naïve CD4 T cells presented a significantly higher proportion in the low-risk group (p<0.05) while M1 macrophages, CD8 T cells, resting mast cells, activated and resting CD4 memory T cells manifested a higher infiltration in the high-risk group (p<0.05). A positive correlation with the risk score was found in infiltration of M1 macrophages, CD8 T cells, and resting CD4 T cells, while a negative correlation was identified in infiltration of CD4 naïve T cells, monocytes, and activated mast cells (**Figure 7B**). HLA family proteins were also found to be significantly higher in the high-risk group (**Figure 7C**). Immune checkpoint proteins including LAG3, CTLA4, HAVCR2, PDCD1, and CD274 also showed significantly higher expression in the high-risk group than in low-risk group (**Figure 7D**).

Research indicates therapeutic effects for anti-PD1 and anti-CTLA4 therapies in glioma (19). We therefore explored possible correlations of the m5C LPS with anti-PD1 and anti-CTLA4 therapies using a subclass mapping algorithm (20). We compared the expression matrix of high and low risk groups to that of a melanoma dataset in which patients underwent anti-PD1 and anti-CTLA4 therapies (17). High risk group patients were generally more responsive to anti-CTLA4 (nominal p value = 0.012) and anti-PD1 therapies (nominal p value = 0.14) (**Figure 7E**).

### Differential Somatic Mutations and Copy Number Variations in Different Risk Groups

**Figure 8:**
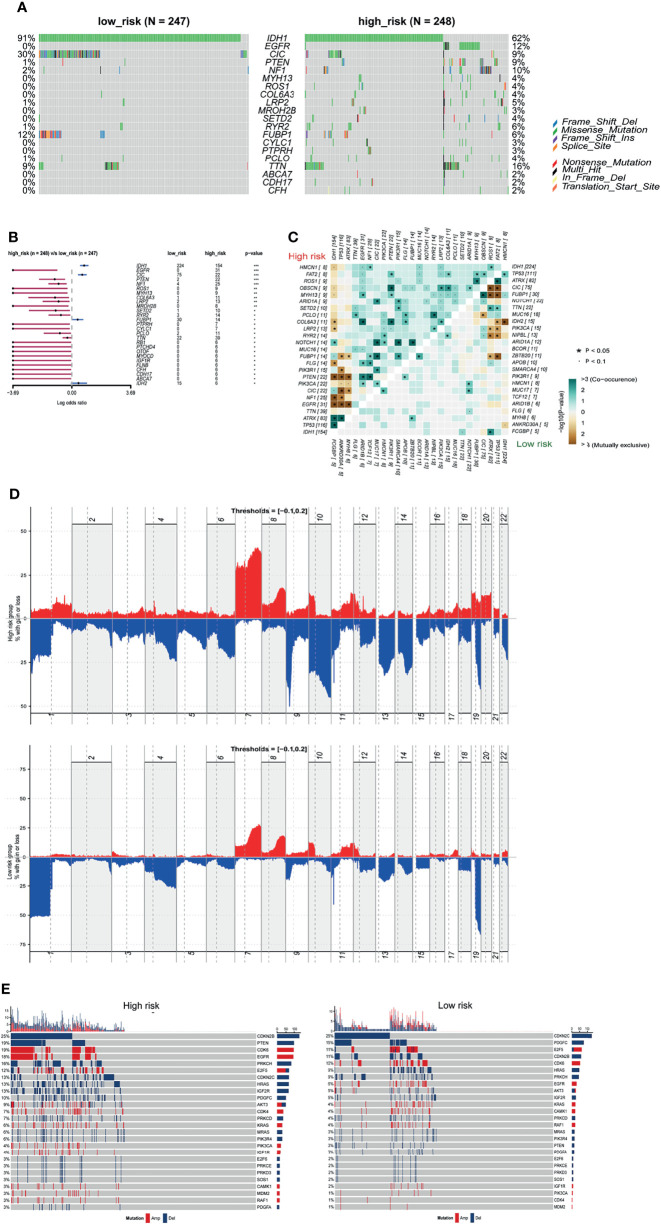
Somatic mutation and CNVs in different risk groups. **(A)** Seven significantly different mutated driver genes between high and low risk groups. **(B)** Forest plot of differentially mutated genes between high and low risk groups. **(C)** Heatmap of mutually exclusive and co-occurrent mutated genes in high and low risk groups. **(D)** Distribution of copy number variations in high and low risk groups. **(E)** Differential CNVs of genes in high and low risk groups.

The 2016 WHO classification of CNS tumors incorporated molecular characteristics including somatic mutations and copy number variations (CNVs) with histological features for a summed-up diagnosis. We analyzed the somatic mutations and CNVs in the current study and explored their relationship with the m5C LPS risk groups. We compared the top differentially mutated genes in the high and low risk groups. IDH1, CIC, and FUBP1 had higher mutation rates in the low-risk group while EGFR, NF1 and PTEN showed higher mutation rates in the high-risk group (**Figures 8A, B**). We also investigated cooccurrence and mutually exclusive genes in the risk groups. EGFR, PTEN and NF1 showed mutual exclusivity with IDH1, TP53 and ATRX in the high-risk group. Cooccurrence was observed in CIC with IDH1, PTEN with EGFR and NF1, and FUBP1 with NF1 and CIC in the high-risk group (**Figure 8C**). We also performed GISTIC2 to identify driver CNVs in different risk groups (18). The high-risk group presented an overall higher rate of copy number amplification and deletion with the exception that deletion on 1p and 19q was observed predominantly in the low-risk group. Deletions on Chr 9p, 10, 13q and 14q and amplifications on Chr 7, 19, 20 were observed mainly in the high-risk group (**Figure 8D**). Distribution of CNVs in different genes between the two risk groups were also analyzed (**Figure 8E**). CDKN2B, PTEN, CDK6, EGFR, PRKCH, and IGF2R showed higher CNV rates in the high-risk group while CDKN2C and PDGFC showed higher CNV rates in the low-risk group.

### m5C-Related LPS in Prediction of Chemotherapeutics Response

**Figure 9:**
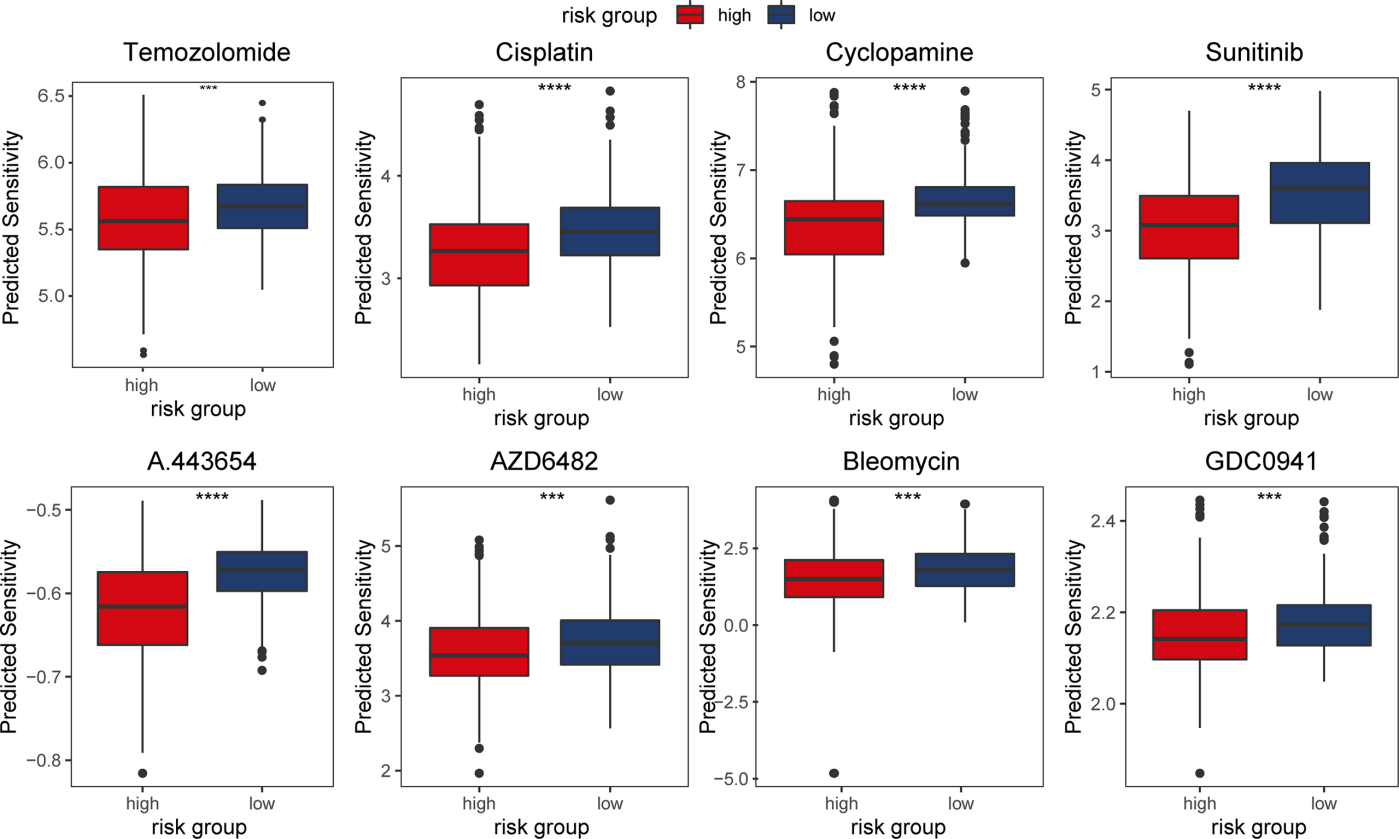
Predicted response of patients in TCGA dataset to chemotherapeutic agents in different risk groups.

Temozolomide has been the first line treatment after surgery for gliomas for some time (21). However, with vast differences in response to temozolomide in patients, researchers have been investigating novel chemotherapeutics for glioma (22, 23). In light of the aforementioned findings in enriched pathways and CNVs in the m5C LPS high risk group, we used the ‘pRRophetic’ package to predict response of patients to chemotherapeutics in different risk groups. We selected Temozolomide, Bleomycin, Cisplatin, Cyclopamine, A.443654 (Akt inhibitor), AZD6482(PI3K inhibitor), GDC0941(PI3K inhibitor), and metformin to analyze levels of response in different risk groups. Results revealed a lower estimated IC50 in the high-risk group for the chosen drugs (**Figure 9**).

## Discussion

Unlike the neurons that are terminally differentiated cells, astrocytes still retain the ability to self-renew and proliferate, which can potentially transform to cancer cells under pathological conditions. This may be attributed to the different effects of abnormal activation of intracellular pathways in neurons and glial cells in the brain (24). Previous studies indicated that the most common histological type of primary CNS tumor is glioma, which is derived from glial cells of astrocytic, oligodendroglia and ependymal origin, with an annual incidence of 5-6/100,000 individuals worldwide (25, 26). Our previous study presented a novel integrated system for glioma individualized therapeutics modeling and screening (27). Studies have also reported predictive models of tumor prognosis and therapeutics using bioinformatics methods (9, 22).

In the current study we identified the m5C-related LPS containing eight lncRNAs, two of which have been reported in the literature. LINC00632 was reported to be associated with several tumors including multiple myeloma cell drug resistance (28), and melanoma invasion and metastasis (29). PAXIP1-AS2 is involved in endometrial cancer development (30). Univariate and multivariate Cox regression analyses with other clinicopathological factors confirmed the risk score to be an independent prognostic factor for LGG patients. The robust predictive performance of the m5C LPS was also confirmed *via* ROC curves in differential clinicopathological subgroups. Many studies have implicated plasma/serum lncRNAs with tumor initiation and diagnosis (31, 32). The current study is the first to construct a m5C-related lncRNA signature in lower grade glioma. The m5C LPS presented a good prognostic ability with one-, three-, and five- year AUC being 0.873, 0.865, and 0.772 respectively compared to existing signatures with AUC ranging from 0.741 to 0.901 (**Supplementary Table 2**) (33–36). The m5C LPS also correlated significantly with immune cell infiltration, tumor mutation, and copy number variation. The m5C LPS could be utilized in diagnosis, drug response and prognosis prediction of lower grade glioma.

LncRNAs have been reported to participate in tumor initiation and progression through reprogramming tumor microenvironment. LINC00665 was reported to affect the infiltration of macrophages and DCs, suppress Tregs, and prevent T cell exhaustion. LncRNA TCL6 is related to tumor infiltrating lymphocytes and immune checkpoint molecules including PD1, PDL1 and CTLA4. Multiple lncRNAs are reported to regulate macrophage polarization and their protein secretion (37). The current study identified T cell differentiation, interferon gamma and alpha signaling pathways to be enriched in the high-risk group. M1 macrophages, CD8 T cells, resting mast cells, activated and resting CD4 memory T cells were found to be highly infiltrated in the high-risk group. Infiltration of M1 macrophages, CD8 T cells and resting CD4 T cells was found to be positively correlated with the risk score while infiltration of CD4 naïve T cells, monocytes and activated mast cells was negatively correlated with the risk score. Differential infiltration of various immune cells in the two risk groups may be the result of the overall difference between m5C LPS high and low risk groups or the direct effect of m5C lncRNAs differential expression. HLA proteins play important roles in antitumor immunity through maintaining CTL antigen presentation and modulating NK cell function. We thus evaluated the expression of HLA proteins in different risk groups. Expression of HLA and immune checkpoint proteins was significantly higher in the high-risk group. Response to immune checkpoint inhibitors in both risk groups was explored using a subclass mapping algorithm. The high-risk group was found to be more responsive to anti-CTLA4 therapy. The mechanism of correlation between m5C lncRNAs and immune infiltration/response are to be further explored.

We also evaluated somatic mutation, CNVs, and response to chemotherapeutic agents in different risk groups. IDH1, CIC, NIPBL, and FUBP1 showed significantly higher mutation rates in the low-risk group while EGFR, NF1, and PTEN showed higher mutation rates in the high-risk group. Deletion on 1p and 19q was observed predominantly in the low-risk group. These results conform to reports of better prognosis in patients with IDH1 mutation or 1p19q codeletion. In predicting chemotherapeutics response, we investigated agents including Akt inhibitor A.443654, PI3K inhibitor AZD6482 and GDC0941 targeting pathways indicated in GSEA analysis. Results showed that cases in the high-risk group were more responsive to the drugs. Mechanism of the correlation between m5C LPS and therapeutics response needs further research. Still, *in vitro* validation of drug response experiments is needed to confirm the predictions.

In summary, we report a lncRNA signature that may contribute to stratification of LGG patients in their prognosis and response to immune- and chemotherapies. There are limitations to the current study including a restricted number of cases in the validation set, lack of further *in vitro* and *in vivo* studies into the correlations of m5C related lncRNAs with tumor immune microenvironment, somatic mutation and CNVs. However, the m5C-related lncRNA prognostic signature should stimulate further laboratory research into the specific lncRNAs from which it is composed, as well as potential biomarker research using patient-derived tumor or serum samples.

## Data Availability Statement

The datasets presented in this study can be found in online repositories. The names of the repository/repositories and accession number(s) can be found in the article/**Supplementary Material**.

## Ethics Statement

The studies involving human participants were reviewed and approved by the ethics committee of Xiangya Hospital, Central South University. The patients/participants provided their written informed consent to participate in this study.

## Author Contributions

CL and LZ conceived and designed the study. HSZ, MM, ZW, and HZ performed the data mining and statistical analyses. HSZ drafted the initial manuscript. LZ, CL, and LY made critical comments and revision for the initial manuscript. All authors reviewed and approved the final manuscript.

## Conflict of Interest

The authors declare that the research was conducted in the absence of any commercial or financial relationships that could be construed as a potential conflict of interest.

## Publisher’s Note

All claims expressed in this article are solely those of the authors and do not necessarily represent those of their affiliated organizations, or those of the publisher, the editors and the reviewers. Any product that may be evaluated in this article, or claim that may be made by its manufacturer, is not guaranteed or endorsed by the publisher.

## Supplementary Material

Supplementary Figure 1 Realtime-qPCR validation of relative expression of the eight lncRNAs of m5C-related LPS in 27 LGG samples.

Supplementary Figure 2 Relative expression of m5C LPS lncRNAs in IDH wild type and IDH mutant samples.

Supplementary Figure 3 Relative expression of m5C LPS lncRNAs in MGMT promoter methylated and unmethylated samples.
