# Supplementary figures and images for: The Role of m5C-Related lncRNAs in Predicting Overall Prognosis and Regulating the Lower Grade Glioma Microenvironment
Source: Front Oncol. 2022 Mar 18;12:814742. doi: 10.3389/fonc.2022.814742 (PMC8971304; doi:10.3389/fonc.2022.814742)

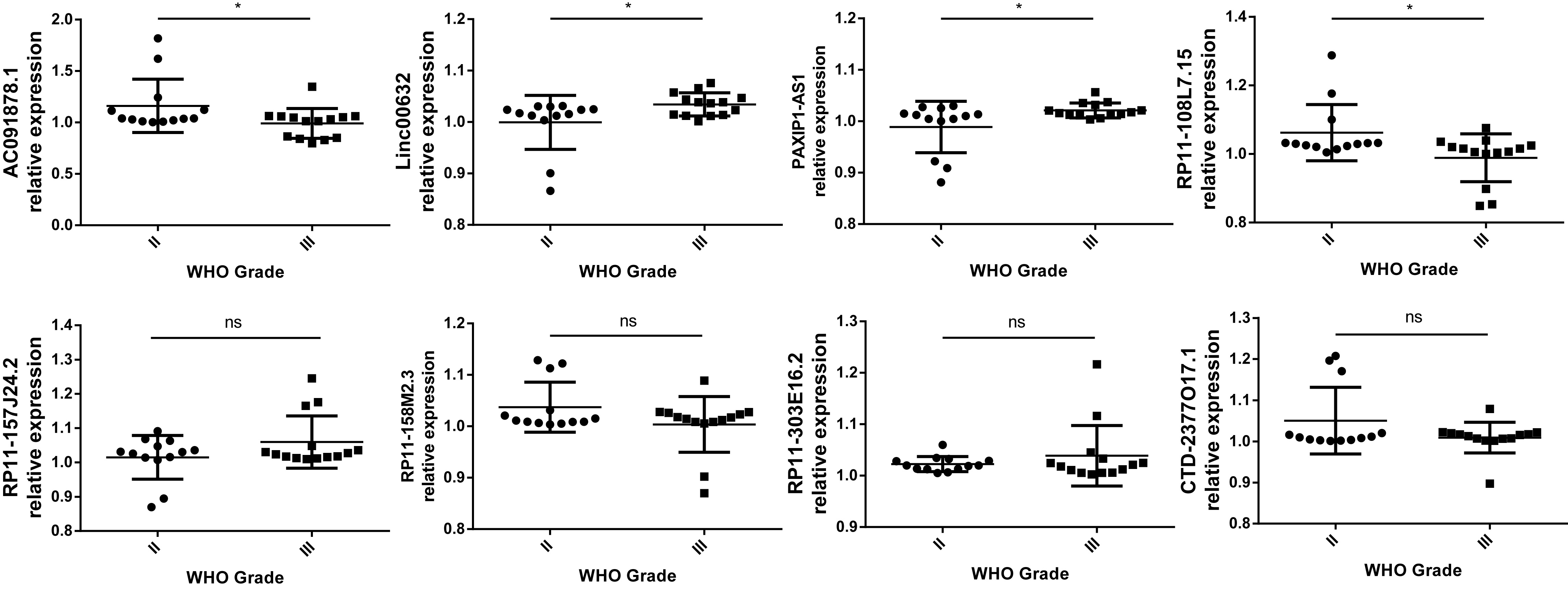

Supplement: Supplementary Figure 1 — Realtime-qPCR validation of relative expression of the eight lncRNAs of m5C-related LPS in 27 LGG samples. [file Image_1.jpeg]

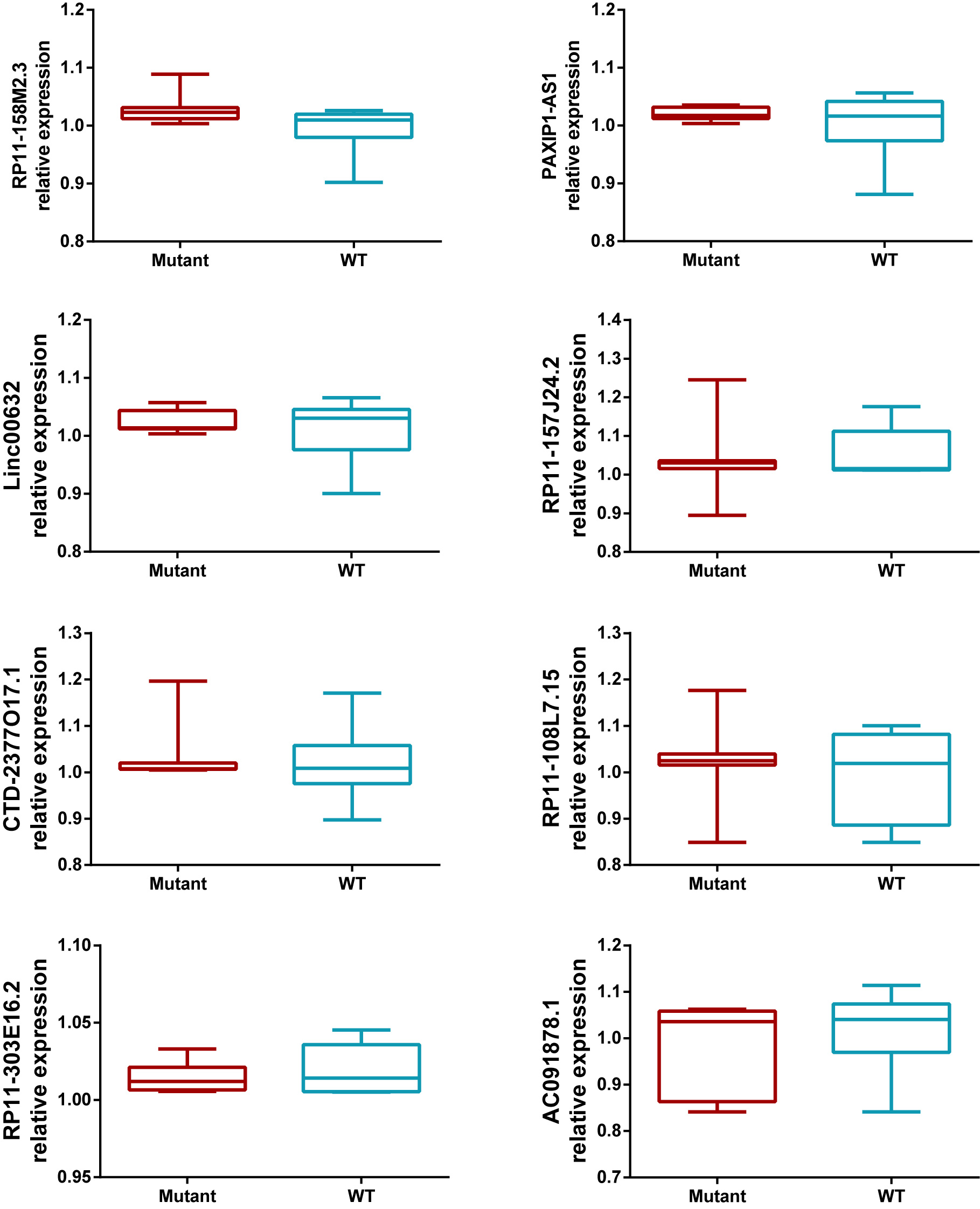

Supplement: Supplementary Figure 2 — Relative expression of m5C LPS lncRNAs in IDH wild type and IDH mutant samples. [file Image_2.jpeg]

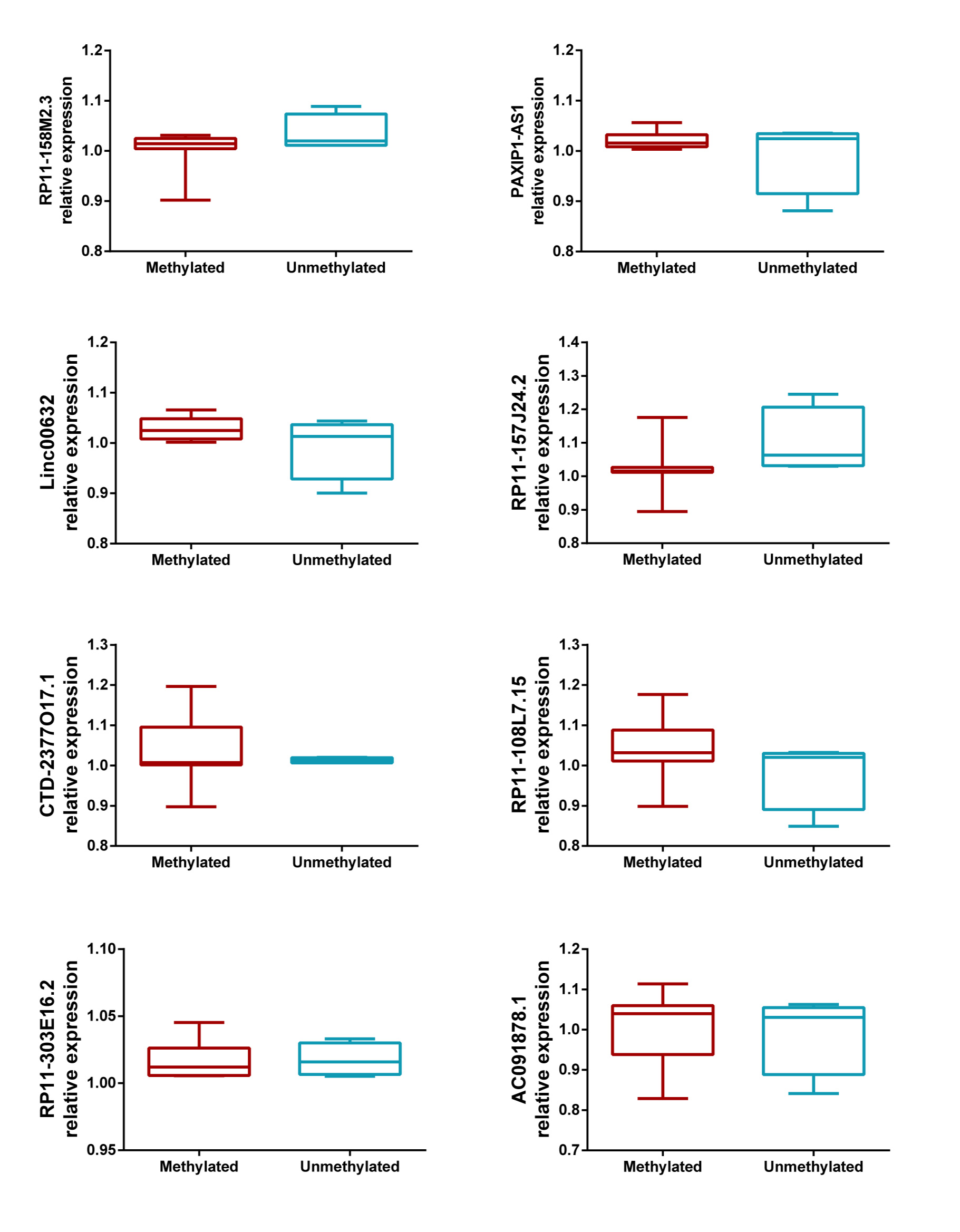

Supplement: Supplementary Figure 3 — Relative expression of m5C LPS lncRNAs in MGMT promoter methylated and unmethylated samples. [file Image_3.jpeg]
