# Supplementary material for: The Role of m5C-Related lncRNAs in Predicting Overall Prognosis and Regulating the Lower Grade Glioma Microenvironment
Source: Front Oncol. 2022 Mar 18;12:814742. doi: 10.3389/fonc.2022.814742 (PMC8971304; doi:10.3389/fonc.2022.814742)
Supplement: Supplementary file 4 [file Table_1.docx]

| lncRNA | Primer |
| --- | --- |
| RP11-157J24.2 | 5’-CCCTTTCACTACAACCCCCG-3’  5’-CCCTCAAACTCTGTGCCTGTT-3’ |
| Linc00632 | 5’-TGGAAGCAGACTTGTGTCCGCA-3’  5’-TGCTTGTCGGTGTGCCTTTGGA-3’ |
| AC091878.1 | 5’-TTGGAGGCTGAGGCAGGAAGAG-3’  5’-CAGGCTGGACTACAGTGGTTTGATC-3’ |
| RP11-303E16.2 | 5’-TTGAATTGGGCCAAATTGCGATGAC-3’  5’-TTGGTGCTGTTGAGGAGTTGTAGTG-3’ |
| RP11-108L7.15 | 5’-TCAACAACAAACTCCACGGACCTAG-3’  5’-GGCACGATTCCTACTACCAGAAGC-3’ |
| CTD-2377O17.1 | 5’-TAGAGCCCGGACTTTCATCTGGAG-3’  5’-GGATGGAACCCTGTTGTCTTTCTGG-3’ |
| RP11-158M2.3 | 5’-TGCTGATACTTCTGACGCGG-3’  5’-CCCAGTCGCCTGTTTGCAT-3’ |
| PAXIP1-AS1 | 5’-ACTACACAGCGGCGATTCTAATGG-3’  5’-AGGTGTCTTGGGCAGGTGATCC-3’ |
| GAPDH | 5’-AACGGATTTGGTCGTATTGG-3’  5’-TTGATTTTGGAGGGATCTCG-3’ |

**Table S1. Primer sequences for qRT-PCR analysis.**
