# Supplementary material for: The Role of m5C-Related lncRNAs in Predicting Overall Prognosis and Regulating the Lower Grade Glioma Microenvironment
Source: Front Oncol. 2022 Mar 18;12:814742. doi: 10.3389/fonc.2022.814742 (PMC8971304; doi:10.3389/fonc.2022.814742)
Supplement: Supplementary file 5 [file Table_2.docx]

**Table S2. Comparison of prognostic signatures for LGG.**

| Authors | Signature type | AUC in training set | | | AUC in validation set | | |
| --- | --- | --- | --- | --- | --- | --- | --- |
|  |  | 1-year | 3-year | 5-year | 1-year | 3-year | 5-year |
| Wang et al. | 7 autophagy-related genes | 0.901 | 0.848 | 0.750 | 0.830 | 0.828 | 0.755 |
| Lin et al. | 5 hypoxia genes | 0.781 | 0.769 | 0.712 | 0.741 | 0.780 | 0.796 |
| Xu et al. | 13 1p19q codeletion-related genes | 0.896 | 0.785 | 0.708 | 0.750 | 0.793 | 0.818 |
| Li et al. | 7 m6A-related genes | 0.798 | 0.813 | 0.784 | 0.748 | 0.774 | 0.719 |
| Yu et al. | 13 epigenetic-related genes | 0.874 | 0.866 | - | 0.81 | 0.774 | - |
